# Paradoxical Effect of Grape Pomace Extract on Cisplatin-Induced Acute Kidney Injury in Rats

**DOI:** 10.3390/pharmaceutics11120656

**Published:** 2019-12-06

**Authors:** Maria Adriana Neag, Calin Iosif Mitre, Andrei Otto Mitre, Vlad Morhan, Adrian Catinean, Emil Claudiu Botan, Carmen Stanca Melincovici, Dana Maria Muntean, Anca Dana Buzoianu

**Affiliations:** 1 Pharmacology, Toxicology and Clinical Pharmacology Department, Iuliu Hatieganu University of Medicine and Pharmacy, 23. Marinescu Street, 400337 Cluj-Napoca, Romania; borosmeda@yahoo.com (M.A.N.); abuzoianu@umfcluj.ro (A.D.B.); 2 Department of Anaesthesiology and Intensive Care, Iuliu Hatieganu University of Medicine and Pharmacy, 19-21. Croitorilor Street, 400162 Cluj-Napoca, Romania; 3 Faculty of Medicine, Iuliu Hatieganu University of Medicine and Pharmacy, 8. Babes Street, 400012 Cluj-Napoca, Romania; andrei.mitre97@gmail.com (A.O.M.); vmorhan98@gmail.com (V.M.); 4 Department of Internal Medicine, Iuliu Hatieganu University of Medicine and Pharmacy, 3-5. Clinicilor Street, 400006 Cluj-Napoca, Romania; catinean1972@yahoo.com; 5 County Emergency Hospital Cluj-Napoca, 3-5. Clinicilor Street, 400006 Cluj-Napoca, Romania; botanemil@gmail.com; 6 Histology Department, Iuliu Hatieganu University of Medicine and Pharmacy, 4-6. Pasteur Street, 400349 Cluj-Napoca, Romania; carmen.melincovici@umfcluj.ro; 7 Department of Pharmaceutical Technology and Biopharmaceutics, Iuliu Hatieganu University of Medicine and Pharmacy, 12. Ion Creanga Street, 400010 Cluj-Napoca, Romania; dana.muntean@umfcluj.ro

**Keywords:** cisplatin, grape extract, resveratrol, nephrotoxicity

## Abstract

Cisplatin is one of the most used drugs in the therapy of different types of cancer. However, its use is limited by nephrotoxicity. This study investigated the effects of a commercially available grape pomace extract (GE) from *Vitis vinifera* on cisplatin-induced kidney toxicity in rats. Sixty-four male Wistar albino rats were randomly divided into eight groups. Groups 1–3 were controls, receiving 0.9% saline and doses 1 and 2 of GE respectively. Cisplatin was given to groups 4–8. Two groups received pretreatment with GE, while another two groups received pre- and post-treatment with GE. Blood samples were collected and all animals sacrificed. Kidneys were harvested for histopathological analysis. GE significantly increased blood creatinine and urea levels, the severity of kidney histopathological damage, and mortality in all cisplatin groups, except for group 7 which received pre- and post-treatment with a low dose of GE. Renal toxicity was determined by mortality and severe histopathological renal lesions. Additionally, the serum total antioxidant capacity (TAC) was not significantly modified in the treated groups compared to the control. These results indicate that the GE did not have a protective effect on cisplatin-induced nephrotoxicity; on the contrary, GE accentuated the toxic effect of cisplatin.

## 1. Introduction

One of the classical anti-cancer drugs used in solid cancer is cisplatin (cis-diamminedichloroplatinum(II)) (CIS), and this is still commonly used today in numerous oncological treatment regimens. Presenting increased effectiveness with increased dosage, several studies have attempted to reduce the limiting factors of CIS administration, the major one being its inherent nephrotoxicity [1,2,3]. Even though hearing loss, gastro-intestinal problems, and immune suppression are common, these do not pose a limiting factor on platinum salt treatment [4,5].

Despite the fact that we live in the age of targeted cancer treatments and the subsequent unpopularity of classical medications such as antifolates and platinum salts, these are still fairly common in practice and finding ways to make them more effective is still a valid area of research. CIS renal toxicity is generally considered to be influenced by several factors, the most notable being the destruction of renal epithelial cells with subsequent loss of kidney function, damaging effects on mtDNA, and also, the activation of apoptosis and necrosis pathways inside the cell. As of now, different strategies to reduce the side effects have been tried, with different levels of success. In day to day clinical practice, salt-based volume expansion is used with added diuretics such as mannitol or furosemide [6,7,8]. Another noteworthy strategy is the administration of Amifostine to treat ovarian cancers, with its protective mechanism still being a debated topic in literature [9,10,11]. Nevertheless, the incomplete success of these approaches has opened the way for combined treatments and the proposal of a few possible new candidates. Our study focuses on one such candidate, a natural grape-based extract from the market.

Numerous in vivo studies have indicated the antioxidant and protective effects of grape juice or grape seed extract, however, the information regarding the protective role of grape pomace extract is limited [12,13,14].

Of the numerous compounds with theorized nephroprotective effects, resveratrol and polyphenols such as phenolic acids present the most promise [15]. In vivo experimental studies have provided evidence of the nephroprotective effect of resveratrol (a major component in grape juice) on the toxicity induced by drugs or toxins (gentamicin, cyclosporine, cisplatin, arsenic trioxide). The protective mechanisms are mainly related to oxidative stress reduction and nitric oxide modulation [16,17,18,19].

This study investigated the dose–response effect of grape pomace extract (GE) on CIS-induced renal changes in rats.

## 2. Materials and Methods

### 2.1. Animals

The animals used in this study were Charles River Wistar (*n* = 64) white male rats with a median weight of 265 ± 15 g. The animals were housed in polysulfone type III-H open-top cages (Tecniplast, Buguggiate, Italy) and had access to filtered tap water in bottles and pelleted feed (Cantacuzino Institute, Bucharest, Romania) ad libitum. The bedding was a standard wood chip aseptic bedding (LignocelVR; J. Rettenmaier & SohneGmBH Co. KG, Rosenberg, Germany). The rats were acquired from the Laboratory Animal Facility of the Iuliu Hatieganu University of Medicine and Pharmacy and were kept at a standard temperature of 20 ± 2 °C and a relative humidity of 55% ± 10%, in a 12:12 h light-dark cycle (lights on from 7 a.m. to 7 p.m.) with a light intensity of 285 lux at 1 m above the floor. The working protocol was revised and approved by the Ethics Committee of the University (no. 112) from 11 May 2018.

Prior to the onset of the study, all animals were quarantined and left to acclimatize to the separation from the rat colony for 2 days. The “Guiding Principles in the Use of Animals in Toxicology”, adopted by the Society of Toxicology (USA) and the National Law regarding the protection of animals used for scientific research, were the specific regulations and amendments from this study.

### 2.2. Grape Extract (GE)

As per the manufacturer’s information, GE contains extracts from grape (*Vitis vinifera*) seeds, rahis and peel (700 mg/mL) in a water-based, homogenous solution. The polyphenolic value of the product is 300 mg gallic acid equivalents (GAE)/mL. For this experiment, we used three bottles of commercially available products, homogenized together in one recipient. Thus, all animals received the same product with the same composition for the entire treatment period, without variability between the active compounds.

We used GE at two different concentrations. The doses were measured in mL/kg body weight (b.w.) and also in mg GAE. The first dose consisted of 0.35 mL/kg b.w. or 105 mg GAE (GEd1), and the second dose was 0.7 mL/kg b.w. or 210 mg GAE (GEd2).

### 2.3. Experimental Design

**Figure 1:**
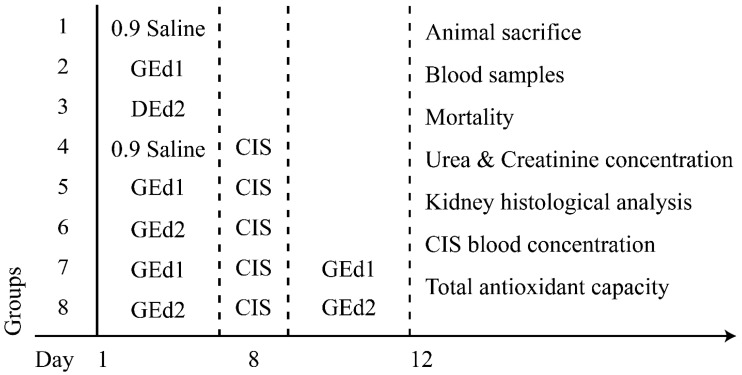
Experimental protocol and administered substances. Abbreviations are as follows: GEd, grape extract dose (1, 0.35 mL/kg b.w.; 2, 0.7 mL/kg b.w.); CIS, cisplatinum.

A total of 64 rats were randomly divided into eight groups (*n* = 8). In the first 7 days of treatment, each animal received treatment, according to its group. Groups 1 and 4 received 0.9% saline solution, groups 2, 5, and 7 received GEd1, and groups 3, 6, and 8 received GEd2. On the eighth day, groups 4–8 received one intraperitoneal dose of 7.5 mg/kg b.w. CIS. On the next 4 days, groups 7 and 8 received GEd1 or GEd2, according to their previous treatment. On the last day of the study, blood samples were collected from the retroorbital sinus plexus of the rats under mild anesthesia, and then all animals were euthanized using deep anesthesia. Kidneys were removed for the histopathological analysis. After coagulation, the serum was separated by centrifuging at 4000 rpm for 15 min and was kept at −20 °C until further biochemical analysis. (Figure 1)

### 2.4. Measurements

We measured the mortality percentage for each group. We determined the urea and creatinine levels using spectrophotometry. The CIS plasma concentrations were estimated by using a validated high-throughput liquid chromatography (HPLC) (Agilent Technologies, Santa Clara, CA, USA) analytical method [20]. A 3 × 100 mm, 3.5 µm Zorbax C 18 chromatographic column (Agilent Technologies) was used for separation. The mobile phase consisted of 80% methanol and 20% water. The flow rate was 1.5 mL/min; the thermostat temperature was set at 25 °C. The peaks were detected by using an Agilent 1100 series UV detector (at 254 nm). The calibration curve was linear over a concentration range of 1–10 µg/mL.

The total antioxidant capacity (TAC) of the GE in vitro and in vivo (after animal administration) was measured according to the method previously described by Erel [21].

### 2.5. Histopathological Examination

At the end of the study, the kidneys of each animal were removed. Sections of excised kidney tissues from all animals were preserved in 10% formaldehyde, dehydrated in graduated ethanol, and incorporated into paraffin. Thereafter, sections of the liver were cut at 7 μm on a microtome (Leica RM 2145), (Leica Microsystems, Wetzlar, Germany) and mounted on glass slides. Slides were stained with hematoxylin-eosin for histological evaluation. The histological sections were examined under a Leica DM750 microscope, and the images were captured using a Leica ICC 50 HD camera (Leica Microsystems) connected to the microscope.

We determined the renal parenchymal damage with a score based on the tubular necrosis percentage observed in the tissues (0—no damage; 1—isolated unicellular necrosis; 2—tubular necrosis <25%, 3—tubular necrosis between 25% and 50%; 4—tubular necrosis >50%).

### 2.6. Statistical Analysis

The statistical analysis was done using SPSS 18.0 software (IBM, Armonk, NY, USA). One-way analysis of variance (one-way ANOVA) followed by post hoc testing was performed to detect the differences between groups. *p* < 0.05 was considered statistically significant.

## 3. Results

### 3.1. Mortality

The mortality results are presented in Table 1. Control groups had no deaths in the group, while in the CIS group, 50% of the animals died before the end of the experiment. As a trend, the treated groups had high mortality rates, with the only exception being the group that received pre- and post-treatment with the first dose of CIS (GEd1 + CIS + GEd1).

The high mortality in group 8 (GEd2+CIS+GEd2) influenced the following tests, so we did not include the samples from this group in the final statistical analysis.

### 3.2. Urea and Creatinine Levels

**Figure 2:**
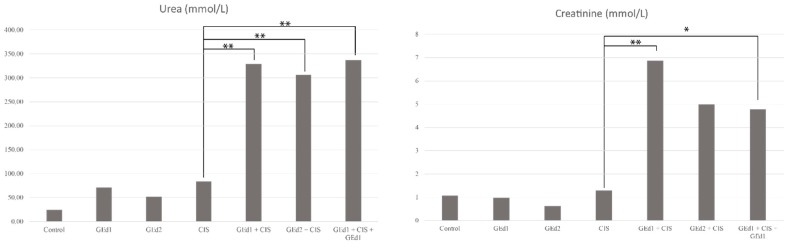
Mean levels of urea and creatinine in the study groups. Abbreviations are as follows: GEd, grape extract dose; CIS, cisplatinum. Statistically significant differences are indicated by asterisks: ** *p* ≤ 0.001, * *p* < 0.05.

Plasmatic urea and creatinine levels were measured for 7 groups and are displayed in Figure 2. Measured values were higher in all groups receiving the grape extract, regardless of the dosage or treatment scheme.

### 3.3. Kidney Histology Examination

The scores according to the degree of tubular necrosis are summarized in Table 2.

**Figure 3:**
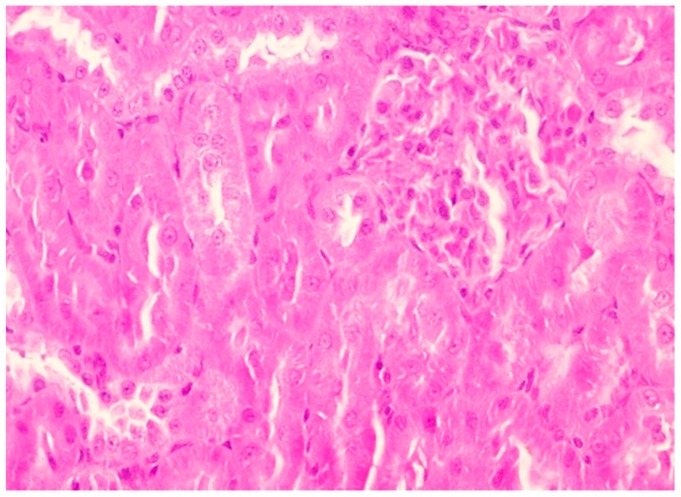
Renal histological aspect of the control group (group 1). Description: Normal kidney histological aspect with slight congestion (HE, 400×).

Renal tissue with normal architecture was observed in the control group (Figure 3).

**Figure 4:**
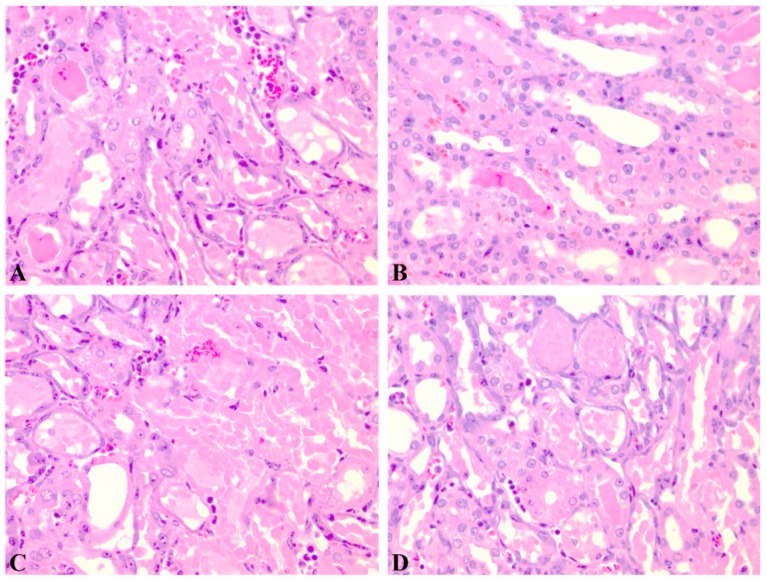
Renal histological aspect of the cisplatin group (group 4). Descriptions: (**A**, **B**, **C**) Partially and complete tubular epithelium necrosis, reactive modifications of the remaining epithelium, hyaline cylinders in the medulla (HE, 200×); (**D**) reactive epithelium and hyaline cylinders (HE, 200×).

**Figure 5:**
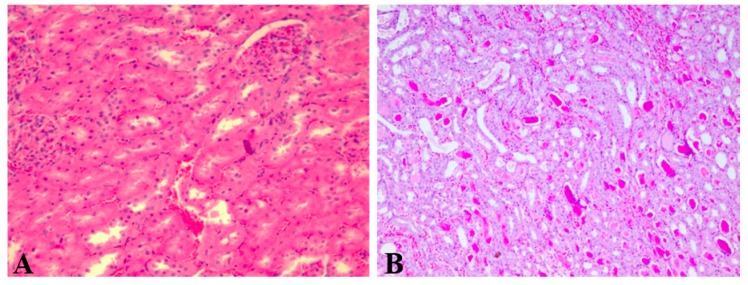
Renal histological aspect of groups receiving grape extract only before cisplatin (groups 5 and 6). Descriptions: (**A**) Group 5, GEd1 + CIS—marked stasis in the medulla, moderate stasis in the glomeruli, frequent intratubular hyaline cylinders in the cortical and medullar area, predominantly in the collector tubules, and tubular necrosis (15%) (HE, 100×); (**B**) Group 6, GEd2 + CIS—hyaline cylinders in the medulla and cortical area (HE, 50×).

**Figure 6:**
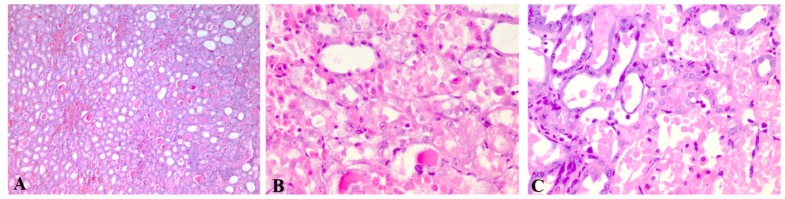
Renal histological aspect of groups receiving grape extract before and after cisplatin (groups 7 and 8). Descriptions: (**A**) Group 7, GEd1 + CIS + GEd1—interstitial congestion and hyaline cylinders (HE, 50×); (**B**) Group 7, GEd1 + CIS + GEd1—tubular epithelium dystrophy (HE, 200×); (**C**) Group 8, GEd2 + CIS + GEd2—tubular necrosis (HE, 200×).

The results of the histological analysis are displayed in Figure 4, Figure 5 and Figure 6. Extensive degeneration of the tubular epithelium, hyaline casts, interstitial edema, and inflammatory cell infiltration were observed in all groups that received CIS. The mildest tubular necrosis was observed in the group treated with GEd1 before and after the CIS dose.

### 3.4. CIS Plasmatic Concentration

**Figure 7:**
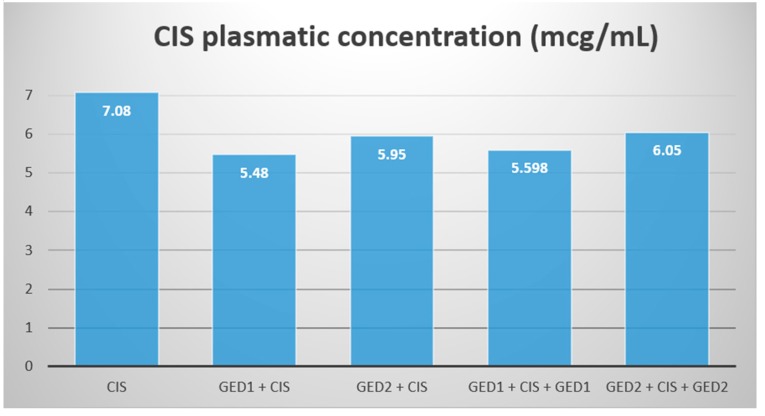
CIS means the plasmatic concentration in the study groups. Abbreviations are as follows: GEd, grape extract dose; CIS, cisplatinum.

The results for CIS plasmatic concentration did not differ significantly for any of the groups (Figure 7).

### 3.5. Total Antioxidant Capacity (TAC)

TAC results were measured in mmol Trolox Equivalents/Liter (TR Eq/L). For the GE, the TAC level was 439.38 mmol TR Eq/L. In the animals, there was no statistically significant difference between the groups. TAC values were lower in the CIS group (Table 3).

## 4. Discussion

The ability of CIS to inhibit cancer growth relies on DNA damage and oxidative stress [22]. The main mechanism of CIS is its ability to inhibit DNA synthesis, cause inter- and intra-strand DNA damage, and repair processes in fast-growing cells [5,23,24].

Furthermore, metals in general and, specifically, platinum compounds are able to generate reactive oxygen species (ROS), which increase its effectiveness and tumor toxicity [25].

The major drawback of CIS treatment is its high toxicity, which mostly damages the heart and kidneys. CIS enters these tissues where it accumulates and produces its cytotoxic effects via oxidative stress damage, inflammation, and apoptotic pathway activation, leading, in the end, to renal and cardiac failure [26,27,28]. CIS will disrupt the mitochondrial membrane both directly—via CIS binding to its proteins, causing mitochondrial dysfunction and cellular death—and also indirectly—by an ROS-mediated reaction. The subsequent mitochondrial damage intensifies the oxidative stress and cell inflammation [29].

This shows that the main role in CIS toxicity is the formation of ROS and oxidative stress [30]. Antioxidants have proven to be a good option against ROS damage and CIS nephrotoxicity, especially when given before CIS treatment [31,32]. In our study we determined the TAC. The minimum mean value was observed in the CIS group. This value was 37.5% lower than the mean value of the control group. So, CIS has pro-oxidant capacity. Grape extracts are a known antioxidant dietary supplement, aimed at opposing the effects of ROS [33,34,35]. Regarding such treatments in CIS toxicity, improvements of the renal function of animal models was observed, but at this point, no clear conclusion regarding its beneficial effect has been made [36,37]. GE increased the antioxidant capacity, but probably not enough to protect against the overall damage of the chemotherapeutic agent. As mentioned above, there are many mechanisms involved in the side effects of CIS. Moreover, the small number of remaining animals after CIS administration made data interpretation difficult.

In previous studies, the dosages of CIS used in single administration varied from 5 to 10 mg/kg b.w. [38,39]. The dose used in our study (7.5 mg/kg b.w.) represents the lethal dose 50 of CIS, and is similar to what Yang et al. reported in their study on female rats [40].

An unexpected event in our study was the high mortality rate of the animals in the treated groups. In all but one group, the mortality percentages were higher than in the CIS group alone. The lowest mortality rate was in the GEd1-treated group before and after CIS (group 7), while the highest mortality rate was in the GEd2-treated group before and after CIS (group 8). For the other two groups, which were only pre-treated with GEd1 and GEd2, the mortality rate was intermediate but was higher than that of the group treated with CIS alone. These results highlight an increase in CIS toxicity by the GE.

The mean creatinine and urea values in the control group were statistically significantly different from the average values of the groups treated with CIS and GE (*p* < 0.05). These results are similar to those obtained in previous studies on CIS, but the addition of GE to the CIS treatment had unexpected negative results [41].

Nephrotoxicity was also revealed by the histopathological examination. In CIS and CIS with GE, severe and extensive degeneration of the tubular epithelium, hyaline castings, interstitial edema, and inflammatory cell infiltration were observed. Tubular necrosis was more important in the groups that received pre-treatment with GEd1 and pre- and post-treatment with GEd2.

Similar to the previous results, the group receiving GEd1 before and after CIS had the lowest tubular necrosis (with patchy, isolated, unicellular necrosis). No histological changes were observed in the groups with GE without CIS. Therefore, nephrotoxicity depends on the interaction between CIS and GE and also on the dose of GE.

Renal CIS uptake is mediated by two membrane transporters, copper transporter 1 and human organic cation transporter 2, which take part in the nephrotoxicity mechanism by stimulating the accumulation of CIS in the renal tissue where it exhibits the stimulation of ROS and oxidative stress [42,43,44,45]. It is known that the CIS concentration in the proximal convoluted tubule can be up to 5 times greater than in the blood and that the concentration in the kidneys is proportional to the concentration in the blood [46].

We hypothesised that the negative outcome of the GE treatment was associated with an increase in the renal uptake of CIS. For this, we analyzed the plasmatic CIS concentration to see if there was a concentration change between the treatment groups. Our results showed no significant difference between the plasmatic concentrations of CIS and only a slight decrease for the GE treated groups.

We therefore conclude that GE did not influence the renal intake of CIS and that there is no pharmacokinetic interaction between CIS and the GE.

In order to establish the cause of the high mortality, we examined the possibility that the GE itself might have had a harmful effect. As mentioned before, GE has a potent antioxidant effect. However, previous studies linked GE with pro-oxidant and DNA-damaging properties [47]. Most of the GE effects are attributed to its rich composition of polyphenols, from which resveratrol is the most abundant and of high importance [48]. Resveratrol was previously linked to a better outcome in CIS-treated animals and is a potent antioxidant [15]. Besides its beneficial effects, resveratrol has also been reported as having pro-oxidant and cytotoxic effects. Additionally, gallic acid, another compound of GE, also exhibits pro-oxidant effects [49].

The TAC results for GE showed a theoretically high free radical scavenging potential and a reduction in oxidative stress. However, the in vivo TAC results showed no significant difference in the redox states of the different studied groups.

Natural products rich in polyphenolic compounds were initially studied initially in vitro and then, some of them were studied in vivo. It has been observed that the in vivo effects do not always correlate with the in vitro ones [50]. As previously mentioned, the main active beneficial compounds of GE are polyphenols. When characterized in vitro, polyphenols present antioxidative properties that translate into potential health benefits. This changes in vivo, because of the modifications caused by the gastrointestinal tract (by the gastrointestinal enzymes and microflora) and by the liver metabolism [51].

The data of our study showed that the in vitro antioxidant capacity of pomace GE could not be translated to in vivo antioxidant capacity. The TAC values of the GE groups were not significantly different compared with those of controls (Table 3). A gap between the in vitro and in vivo antioxidant capacity results of grape extracts has been previously observed in other studies [52].

All being considered, we believe that the toxicity mechanism is not linked to the pro-oxidant effect of these substances, but rather to their DNA-damaging effect.

The exact mechanism of action remains unknown, but a possible toxicity pathway involves the interaction of these substances from the GE with copper ions. This would lead to the synthesis of DNA damaging molecules and also to reactive oxygen radicals and ROS [53].

On the last electron-shell of the copper ion, there is only one electron present, which makes it vulnerable to electron-exchanging reactions in the body. The same is true for platinum, with only one electron on its last electron-shell. This means the toxic effect was most likely caused by the interaction of resveratrol from the GE with the platinum ions in CIS that accumulated in the kidney. This might have resulted in toxic resveratrol radicals that caused DNA cleavage and thus, the induction of cell death.

## 5. Limitations

It is necessary to take into account certain limitations of our study:

1. The small number of animals;
2. The lack of blood samples from the animals that died during the experiment;
3. The missing parameters determined directly from the kidneys;
4. The fact that, in natural extracts, it is very unlikely to have the same individual polyphenol concentration and we did not conduct a phytochemical determination of the administered GE homogenate.

## 6. Conclusions

These results indicate that, despite promising antioxidant and protective theoretical effects, pomace grape extract did not have a protective effect on cisplatin-induced nephrotoxicity. This data further supports the disparity between the in vitro and in vivo actions of pomace grape extract. Moreover, this extract interacted with cisplatin and accentuated its toxic effect. Further investigations are needed to determine the mechanism and the exact substances through which the grape extract increases cisplatin-induced nephrotoxicity.

## Figures and Tables

**Table 1:** Mortality percentages in the study groups.

Group	Mortality (%)
Control	0
GEd1	0
GEd2	0
CIS	50
GEd1 + CIS	62.5
GEd2 + CIS	75
GEd1 + CIS + GEd1	37.5
GEd2 + CIS + GEd2	87.5

Abbreviations are as follows: GEd, grape extract dose; CIS, cisplatinum.

**Table 2:** Tubular necrosis scores in the study group.

Group	Score
Control	0
GE1	0
GE2	0
Cis	2
GE1 + Cis	2
GE2 + Cis	3
GE1 + Cis + GE1	1
GE2 + Cis + GE2	3

Abbreviations are as follows: 0—no damage; 1—patchy isolated unicellular necrosis; 2—tubular necrosis <25%; and 3—tubular necrosis between 25% and 50%; GEd, grape extract dose; CIS, cisplatinum.

**Table 3:** Total antioxidant capacity (TAC) results in the study groups.

Group	TAC (mmol TR Eq/L)
Control	0.16
GEd1	0.18
GEd2	0.19
CIS	0.1
GEd1 + CIS	0.19
GEd2 + CIS	0.16
GEd1 + CIS + GEd1	0.16
GEd2 + CIS + GEd2	0.14

Abbreviations are as follows: GEd, grape extract dose; CIS, cisplatinum; TR Eq, Trolox equivalents.

## References

[1] Sastry J., Kellie S.J. (2005). Severe neurotoxicity, ototoxicity and nephrotoxicity following high-dose cisplatin and amifostine. Pediatr. Hematol. Oncol..

[2] Arany I., Safirstein R.L. (2003). Cisplatin nephrotoxicity. Semin. Nephrol..

[3] Boulikas T. (1992). Poly(ADP-ribose) synthesis in blocked and damaged cells and its relation to carcinogenesis. Anticancer Res..

[4] Hartmann J.T., Fels L.M., Knop S., Stolt H., Kanz L., Bokemeyer C. (2000). A randomized trial comparing the nephrotoxicity of cisplatin/ifosfamide-based combination chemotherapy with or without amifostine in patients with solid tumors. Invest. New Drugs.

[5] Hartmann J.T., Lipp H.-P. (2003). Toxicity of platinum compounds. Expert Opin. Pharmacother..

[6] Cornelison T.L., Reed E. (1993). Nephrotoxicity and hydration management for cisplatin, carboplatin, and ormaplatin. Gynecol. Oncol..

[7] Lehane D., Winston A., Gray R., Daskal Y. (1979). The effect of diuretic pre-treatment on clinical, morphological and ultrastructural cis-platinum induced nephrotoxicity. Int. J. Radiat. Oncol. Biol. Phys..

[8] Al-Sarraf M., Fletcher W., Oishi N., Pugh R., Hewlett J.S., Balducci L., McCracken J., Padilla F. (1982). Cisplatin hydration with and without mannitol diuresis in refractory disseminated malignant melanoma: A southwest oncology group study. Cancer Treat. Rep..

[9] Hensley M.L., Hagerty K.L., Kewalramani T., Green D.M., Meropol N.J., Wasserman T.H., Cohen G.I., Emami B., Gradishar W.J., Mitchell R.B. (2009). American Society of Clinical Oncology 2008 clinical practice guideline update: Use of chemotherapy and radiation therapy protectants. J. Clin. Oncol..

[10] Castiglione F., Dalla Mola A., Porcile G. (1999). Protection of normal tissues from radiation and cytotoxic therapy: The development of amifostine. Tumori.

[11] Capizzi R.L. (1999). Amifostine reduces the incidence of cumulative nephrotoxicity from cisplatin: Laboratory and clinical aspects. Semin. Oncol..

[12] Hansen A.S., Marckmann P., Dragsted L.O., Finné Nielsen I.L., Nielsen S.E., Grønbæk M. (2005). Effect of red wine and red grape extract on blood lipids, haemostatic factors, and other risk factors for cardiovascular disease. Eur. J. Clin. Nutr..

[13] Jayaprakasha G.K., Selvi T., Sakariah K.K. (2003). Antibacterial-and-antioxidant-properties-of-GSE.pdf. Food Res. Int..

[14] Moreno D.A., Ilic N., Poulev A., Brasaemle D.L., Fried S.K., Raskin I. (2003). Inhibitory effects of grape seed extract on lipases. Nutrition.

[15] Darwish M.A., Abo-Youssef A.M., Khalaf M.M., Abo-Saif A.A., Saleh I.G., Abdelghany T.M. (2018). Resveratrol influences platinum pharmacokinetics: A novel mechanism in protection against cisplatin-induced nephrotoxicity. Toxicol. Lett..

[16] Chander V., Tirkey N., Chopra K. (2005). Resveratrol, a polyphenolic phytoalexin protects against cyclosporine-induced nephrotoxicity through nitric oxide dependent mechanism. Toxicology.

[17] Silan C., Uzun Ö., Çomunoǧlu N.Ü., Gokçen S., Bedirhan S., Cengiz M. (2007). Gentamicin-induced nephrotoxicity in rats ameliorated and healing effects of resveratrol. Biol. Pharm. Bull..

[18] Yu M., Xue J., Li Y., Zhang W., Ma D., Liu L., Zhang Z. (2013). Resveratrol protects against arsenic trioxide-induced nephrotoxicity by facilitating arsenic metabolism and decreasing oxidative stress. Arch. Toxicol..

[19] Do Amaral C.L., Francescato H.D.C., Coimbra T.M., Costa R.S., Darin J.D.A.C., Antunes L.M.G., Bianchi M.D.L.P. (2008). Resveratrol attenuates cisplatin-induced nephrotoxicity in rats. Arch. Toxicol..

[20] Lopez-Flores A., Jurado R., Garcia-Lopez P. (2005). A high-performance liquid chromatographic assay for determination of cisplatin in plasma, cancer cell, and tumor samples. J. Pharmacol. Toxicol. Methods.

[21] Erel O. (2004). A novel automated direct measurement method for total antioxidant capacity using a new generation, more stable ABTS radical cation. Clin. Biochem..

[22] Spitz D.R., Phillips J.W., Adams D.T., Sherman C.M., Deen D.F., Li G.C. (1993). Cellular resistance to oxidative stress is accompanied by resistance to cisplatin: The significance of increased catalase activity and total glutathione in hydrogen peroxide-resistant fibroblasts. J. Cell. Physiol..

[23] O’Grady S., Finn S.P., Cuffe S., Richard D.J., O’Byrne K.J., Barr M.P. (2014). The role of DNA repair pathways in cisplatin resistant lung cancer. Cancer Treat. Rev..

[24] Prestayko A.W., D’Aoust J.C., Issell B.F., Crooke S.T. (1979). Cisplatin (cis-diamminedichloroplatinum II). Cancer Treat. Rev..

[25] Florea A.-M., Büsselberg D. (2011). Cisplatin as an anti-tumor drug: Cellular mechanisms of activity, drug resistance and induced side effects. Cancers.

[26] Dugbartey G.J., Peppone L.J., de Graaf I.A.M. (2016). An integrative view of cisplatin-induced renal and cardiac toxicities: Molecular mechanisms, current treatment challenges and potential protective measures. Toxicology.

[27] Ramesh G., Reeves W.B. (2002). TNF-α mediates chemokine and cytokine expression and renal injury in cisplatin nephrotoxicity. J. Clin. Investig..

[28] Demkow U., Biatas-Chromiec B., Stelmaszczyk-Emmel A., Radzikowska E., Wiatr E., Radwan-Rohrenschef P., Szturmowicz M. (2011). The Cardiac Markers and Oxidative Stress Parameters in Advanced Non-Small Cell Lung Cancer Patients Receiving Cisplatin-Based Chemotherapy. EJIFCC.

[29] Yang Z., Schumaker L.M., Egorin M.J., Zuhowski E.G., Guo Z., Cullen K.J. (2006). Cisplatin Preferentially Binds Mitochondrial DNA and Voltage-Dependent Anion Channel Protein in the Mitochondrial Membrane of Head and Neck Squamous Cell Carcinoma: Possible Role in Apoptosis. Clin. Cancer Res..

[30] Jing X.-B., Cai X.-B., Hu H., Chen S.-Z., Chen B.-M., Cai J.-Y. (2007). Reactive oxygen species and mitochondrial membrane potential are modulated during CDDP-induced apoptosis in EC-109 cells. Biochem. Cell Biol..

[31] Noori S., Mahboob T. (2010). Antioxidant effect of carnosine pretreatment on cisplatin-induced renal oxidative stress in rats. Indian J. Clin. Biochem..

[32] Cetin R., Devrim E., Kiliçoğlu B., Avci A., Candir O., Durak I. (2006). Cisplatin impairs antioxidant system and causes oxidation in rat kidney tissues: Possible protective roles of natural antioxidant foods. J. Appl. Toxicol..

[33] Olaku O.O., Ojukwu M.O., Zia F.Z., White J.D. (2015). The Role of Grape Seed Extract in the Treatment of Chemo/Radiotherapy Induced Toxicity: A Systematic Review of Preclinical Studies. Nutr. Cancer.

[34] Trošt K., Klančnik A., Mozetič Vodopivec B., Sternad Lemut M., Jug Novšak K., Raspor P., Smole Možina S. (2016). Polyphenol, antioxidant and antimicrobial potential of six different white and red wine grape processing leftovers. J. Sci. Food Agric..

[35] Balea Ş.S., Pârvu A.E., Pop N., Marín F.Z., Pârvu M. (2018). Polyphenolic Compounds, Antioxidant, and Cardioprotective Effects of Pomace Extracts from Fetească Neagră Cultivar. Oxid. Med. Cell. Longev..

[36] Ko J.-L., Tsai C.-H., Liu T.-C., Lin M.-Y., Lin H.-L., Ou C.-C. (2016). Differential effects of grape juice on gastric emptying and renal function from cisplatin-induced acute adverse toxicity. Hum. Exp. Toxicol..

[37] Wang C.-Z., Fishbein A., Aung H.H., Mehendale S.R., Chang W.-T., Xie J.-T., Li J., Yuan C.-S. (2005). Polyphenol Contents in Grape-Seed Extracts Correlate with Antipica Effects in Cisplatin-Treated Rats. J. Altern. Complement. Med..

[38] Fujikura T., Yasuda H., Iwakura T., Tsuji T., Anders H.-J. (2019). MDM2 inhibitor ameliorates cisplatin-induced nephropathy via NFκΒ signal inhibition. Pharmacol. Res. Perspect..

[39] Sahu A.K., Verma V.K., Mutneja E., Malik S., Nag T.C., Dinda A.K., Arya D.S., Bhatia J. (2019). Mangiferin attenuates cisplatin-induced acute kidney injury in rats mediating modulation of MAPK pathway. Mol. Cell. Biochem..

[40] Li X., Yang S., Lv X., Sun H., Weng J., Liang Y., Zhou D. (2013). The mechanism of mesna in protection from cisplatin-induced ovarian damage in female rats. J. Gynecol. Oncol..

[41] Kociba R.J., Sleight S.D. (1971). Acute toxicologic and pathologic effects of cis-diamminedichloroplatinum (NSC-119875) in the male rat. Cancer Chemother. Rep..

[42] Ishida S., Lee J., Thiele D.J., Herskowitz I. (2002). Uptake of the anticancer drug cisplatin mediated by the copper transporter Ctr1 in yeast and mammals. Proc. Natl. Acad. Sci. USA.

[43] Pabla N., Murphy R.F., Liu K., Dong Z. (2009). The copper transporter Ctr1 contributes to cisplatin uptake by renal tubular cells during cisplatin nephrotoxicity. Am. J. Physiol. Ren. Physiol..

[44] Ciarimboli G., Ludwig T., Lang D., Pavenstädt H., Koepsell H., Piechota H.-J., Haier J., Jaehde U., Zisowsky J., Schlatter E. (2005). Cisplatin nephrotoxicity is critically mediated via the human organic cation transporter 2. Am. J. Pathol..

[45] Sprowl J.A., Lancaster C.S., Pabla N., Hermann E., Kosloske A.M., Gibson A.A., Li L., Zeeh D., Schlatter E., Janke L.J. (2014). Cisplatin-induced renal injury is independently mediated by OCT2 and p53. Clin. Cancer Res..

[46] Yao X., Panichpisal K., Kurtzman N., Nugent K. (2007). Cisplatin Nephrotoxicity: A Review. Am. J. Med. Sci..

[47] Shao Z.-H., Vanden Hoek T.L., Xie J., Wojcik K., Chan K.C., Li C.-Q., Hamann K., Qin Y., Schumacker P.T., Becker L.B. (2003). Grape seed proanthocyanidins induce pro-oxidant toxicity in cardiomyocytes. Cardiovasc. Toxicol..

[48] Liu F.-C., Tsai H.-I., Yu H.-P. (2015). Organ-Protective Effects of Red Wine Extract, Resveratrol, in Oxidative Stress-Mediated Reperfusion Injury. Oxid. Med. Cell. Longev..

[49] Yoshino M., Haneda M., Naruse M., Htay H.H., Iwata S., Tsubouchi R., Murakami K. (2002). Prooxidant action of gallic acid compounds: Copper-dependent strand breaks and the formation of 8-hydroxy-2′-deoxyguanosine in DNA. Toxicol. Vitr..

[50] Halliwell B. (2008). Are polyphenols antioxidants or pro-oxidants? What do we learn from cell culture and in vivo studies?. Arch. Biochem. Biophys..

[51] Rechner A.R., Kuhnle G., Bremner P., Hubbard G.P., Moore K.P., Rice-Evans C.A. (2002). The metabolic fate of dietary polyphenols in humans. Free Radic. Biol. Med..

[52] Veskoukis A.S., Kyparos A., Nikolaidis M.G., Stagos D., Aligiannis N., Halabalaki M., Chronis K., Goutzourelas N., Skaltsounis L., Kouretas D. (2012). The Antioxidant Effects of a Polyphenol-Rich Grape Pomace Extract in Vitro Do Not Correspond in Vivo Using Exercise as an Oxidant Stimulus. Oxid. Med. Cell. Longev..

[53] de la Lastra C.A., Villegas I. (2007). Resveratrol as an antioxidant and pro-oxidant agent: Mechanisms and clinical implications. Biochem. Soc. Trans..

